# Thrombotic microangiopathy associated with arboviral infection: Report of 3 cases

**DOI:** 10.1371/journal.pntd.0009790

**Published:** 2021-10-14

**Authors:** José Luiz Coelho Júnior, Karla Cristina Petruccelli Israel, Carlos Eduardo Everton Machado, Monique Pereira Rêgo Muniz, Giuseppe Cesare Gatto, Flávio Henrique Soares Barros, Kaile de Araújo Cunha, Marcus Vinícius Guimarães de Lacerda, Precil Diego Miranda de Menezes Neves, Gyl Eanes Barros Silva

**Affiliations:** 1 Department of Nephrology, University Hospital of the Federal University of Maranhão (HUUFMA), São Luís, Brazil; 2 Tropical Medicine Foundation Dr. Heitor Vieira Dourado/State University of Amazonas (UEA), Manaus, Brazil; 3 Nephrology Service, University Hospital of Brasilia, University of Brasília (HUB–UNB), Brasília, Brazil; 4 Nephrology Service, High Complexity State Hospital of Maranhão Dr. Carlos Macieira (HCM), São Luís, Brazil; 5 Nephrology Division, Medical School, University of São Paulo (FM-USP), São Paulo, Brazil; 6 Department of Pathology, Ribeirão Preto Medical School, University of São Paulo (FM-USP), Ribeirão Preto, Brazil; Oregon Health and Science University, UNITED STATES

## Abstract

Dengue fever and chikungunya are viral diseases that have spread rapidly throughout the world in recent decades. The occurrence of complications is well known, including prerenal acute kidney injury (AKI), which is usually thought to be caused by dehydration and fluid loss. Thrombotic microangiopathy (TMA) is an uncommon aggravation of dengue fever and chikungunya, with only a few cases described in the medical literature. The aim of this study is to present 3 cases of TMA associated with arboviral infection. Three patients with clinical history, laboratory test, and kidney biopsy results compatible with TMA were selected for the study, 2 of whom had a serological diagnosis of dengue fever and 1 of chikungunya. The 3 patients were followed up at the Federal University of Maranhão Hospital’s Nephrology Service in 2018. A targeted gene panel sequencing (TGPS) plus multiple to atypical hemolytic uremic syndrome (aHUS) multiplex ligation–dependent probe amplification (MLPA) was performed in 2 of the patients and revealed in the patient 1 a heterozygous pathogenic variant in the gene *THBD*, as well as heterozygous deletions in *CFH*, *CFHR1*, and *CFHR3*. In the patient 2, there were heterozygous pathogenic variant in the genes *CFI* and *CFB*, in addition to heterozygous deletions in the genes *CFHR1* and *CFHR3*. Both received treatment with eculizumab and undergone recovery of renal function. The third patient had TMA not classified as either aHUS or thrombotic thrombocytopenic purpura (TTP); he abandoned the treatment and returned to the service after 2 years for a dialysis emergency. Patients with arboviral infectious disease and changes that suggest TMA should have appropriate support to establish early diagnosis and useful treatment.

## Introduction

Dengue fever and chikungunya are viral diseases that have spread rapidly throughout the world in recent decades. They are transmitted by female mosquitoes, particularly *Aedes aegypti* and less often *Aedes albopictus*. These mosquitoes also transmit yellow and Zika fevers. The modifications of the environment by anthropogenic actions, climate conditions, disorganized urban expansion as well as globalization are some of the factors that have facilitated the development and dissemination of these infectious diseases [1,2].

The occurrence of complications is well known, including prerenal acute kidney injury (AKI), which is usually thought to be caused by dehydration and fluid loss. In fact, kidney alterations associated with arboviral infections have not yet been extensively studied. Descriptions on glomerular alterations are scarce because kidney biopsy is not routinely performed in cases of dengue fever because of the possibility of major bleeding [3]. Thrombotic microangiopathy (TMA) is another possible complication of dengue fever, but it is uncommon, with only a few cases described in the medical literature [4–6]. There have been no reports of atypical hemolytic uremic syndrome (aHUS) associated with chikungunya so far. TMA has been reported in association with a wide range of viral, bacterial, fungal, and parasitic infections, although it is frequently unclear if this is a direct effect of the pathogen, a side effect of treatment, or a trigger that unmasks a latent complement disorder [7]. The available data are mostly heterogeneous, and most information comes from case reports.

In this article, we outline 3 cases of atypical presentation of arboviral infections (2 cases of dengue and 1 case of chikungunya), manifesting as TMA.

## Case presentation

### Case 1

A 19-year-old previously healthy male patient came to the emergency room with a 3-day history of fever, nausea, vomiting, decreased urinary volume, and periorbital edema, in addition to joint pain. Physical examination showed edema in the lower limbs (2+/4+) besides periorbital edema. His blood pressure was 150 × 100 mm Hg, his heart rate was 85 beats per minute (bpm), and his axillary temperature was 37.7°C. He reported a similar clinical picture in the joints of relatives and neighbors during the same period.

The tests showed increased urea and creatinine levels (148 mg/dL and 12 mg/dL, respectively), anemia (hemoglobin 6.9 g/dL), and thrombocytopenia (77,000 platelets/mm^3^). The urinary sediment showed leukocyturia and hematuria (22 to 27/field and 28 to 33/field, respectively), in addition to protein levels of 300 mg/dL. The laboratory reference ranges can be seen in S1 Table. An abdominal ultrasound showed kidneys with increased size with no signs of chronic kidney disease (CKD).

The patient was diagnosed with nephritic syndrome, with evolution suggestive of rapidly progressive glomerulonephritis. Pulse therapy with methylprednisolone was administered at 1 g/day for 3 days, and hemodialysis therapy was initiated. Subsequently, empirical pulse therapy with intravenous cyclophosphamide was introduced at a dose of 1 g/month. Etiologic investigation revealed negative findings for autoimmune diseases (antinuclear antibody, perinuclear, and cytoplasmic antineutrophil cytoplasmic antibodies were all negative). Investigation for viral hepatitis, HIV, and syphilis also revealed negative findings. Nonimmune-mediated microangiopathic hemolytic anemia (peripheral blood schistocytes positive, negative direct Coombs test, haptoglobin 23 mg/dL, and lactate dehydrogenase [LDH] 667 U/L), normal blood complement factor levels, and a positive immunoglobulin M (IgM) immunoassay for chikungunya were documented. A neutralization test was performed, which confirmed the result for chikungunya.

**Fig 1:**
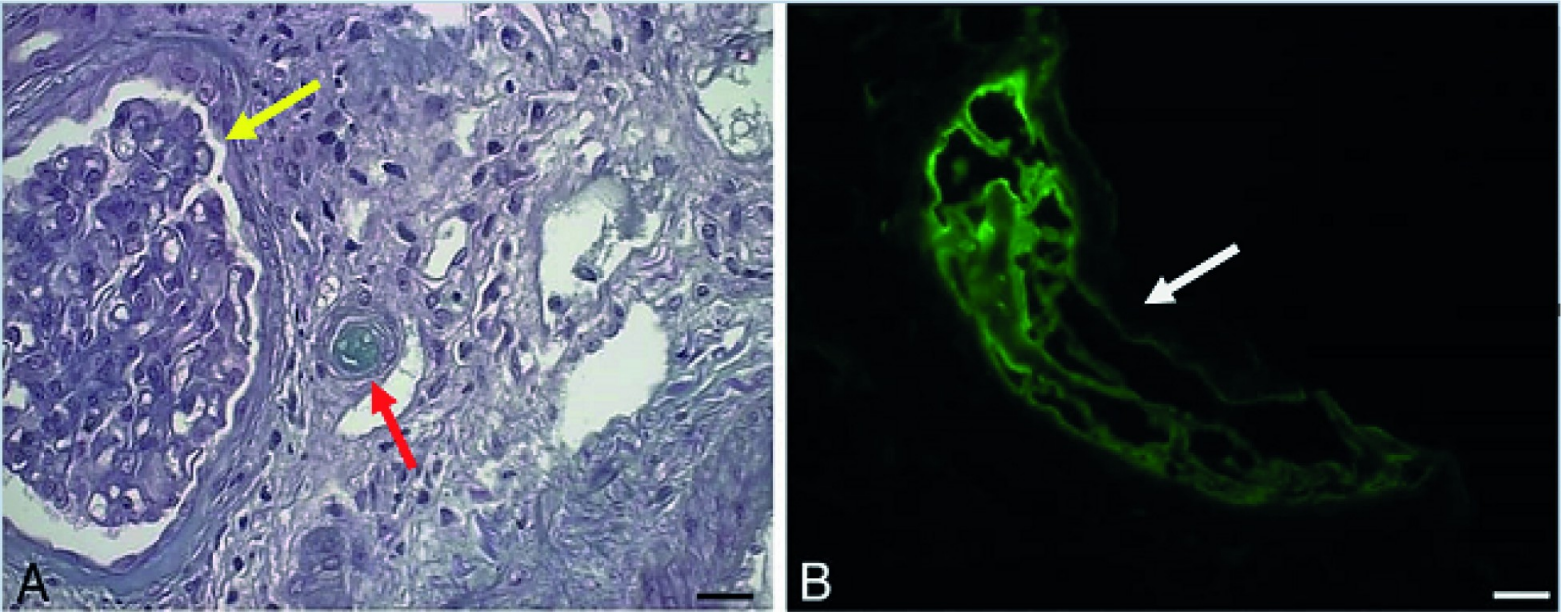
Renal biopsy findings in the patient of the case 1. **(A)** Glomeruli relatively spared (yellow arrow) and an adjacent arteriole with luminal thrombi (red arrow)—Masson’s trichrome. **(B)** Fibrin in arteriole wall detected with antibody to fibrinogen is shown (white arrow)—direct Immunofluorescence. Bars (A, B) = 15 μm.

A kidney biopsy was performed: At light microscopy, all glomeruli presented with mesangiolysis and endothelial edema, some with fibrin thrombi at the vascular pole. Mild interstitial fibrosis and tubular atrophy were observed as well as degenerative tubular changes. Immunofluorescence microscopy was positive for IgM (+3/+3), C3 (+2/+3), and fibrinogen (+3/+3) in vascular walls (Fig 1). Electron microscopy was not performed. The association of histological findings was compatible with TMA.

TMA was investigated, and plasmapheresis was prescribed. Investigation of the ADAMTS13 protease showed moderately reduced enzyme activity (28%), weakening the diagnostic hypothesis of thrombotic thrombocytopenic purpura (TTP). Eculizumab was prescribed 11 days after diagnostic suspicion of aHUS and absence of clinical or laboratory improvement after 5 plasmapheresis sessions. Eculizumab was prescribed as directed by the manufacturer, with an attack dose of 900 mg once a week for 4 weeks, followed by a maintenance dose of 1,200 mg once every 14 days for an indefinite period. A targeted gene panel sequencing (TGPS) + multiplex ligation–dependent probe amplification (MLPA) was performed to investigate a genetic background associated with aHUS, which detected a heterozygous variant c.1208G>A, p.(Arg403Lys) in the gene THBD; a heterozygous deletion of the region downstream of exon 23 in the CFH gene; a heterozygous deletion of all the *CFHR3* gene; and a heterozygous deletion of intron 3 of the *CFHR1* gene, all of which were compatible with increased genetic susceptibility to aHUS. The patient underwent renal replacement therapy (RRT) for 3 months after the introduction of eculizumab, when he presented recovery of kidney function and was discharged from dialysis treatment.

### Case 2

An 18-year-old previously heathy patient, brother of the patient in case 1, presented to the emergency department with dry cough, unmeasured fever, muscle pain, headache, and vomiting that began 4 days earlier. On admission, the patient was hypertensive (150 × 90 mm Hg), icteric (1+/4), and pale (1+/4). The remainder of the physical examination showed no changes. He also presented choluria and abdominal pain, which led to the diagnostic hypothesis of hemorrhagic dengue fever.

Laboratory tests at admission showed hemoglobin 7.0 g/dL, hematocrit 20.5%, 6,370 leukocytes/mm^3^, 99,000 platelets/mm^3^, creatinine 9.98 mg/dL, urea 96 mg/dL, serum potassium 2.9 mEq/L, serum sodium 132 mEq/L, serum albumin 2.26 mg/dL, and prothrombin time 11 seconds. Immunoglobulin G (IgG) and IgM serology tests for dengue were requested; IgG was negative, and IgM was positive. Serology tests for Zika, chikungunya, hepatitis B and C, and HIV were all negative. The patient was referred to HU-UFMA for further investigation and management of his condition. On admission, the patient was hypertensive (150 × 90 mm Hg), icteric (1+/4), and pale (1+/4). The remainder of the physical examination showed no changes. Complementary investigation showed proteinuria, hematuria, leukocyturia, and granular cylinders in the urine: serum LDH 2465 mg/dL, total bilirubin 1.37 mg/mL, indirect bilirubin 0.94 mg/mL, direct bilirubin 0.43 mg/mL, peripheral blood smear positive for schizocytes, negative direct Coombs test, and ADAMTS13 protease >100%.

A kidney biopsy was performed: At light microscopy, all glomeruli presented with mesangiolysis and endothelial edema. Mild interstitial fibrosis and tubular atrophy were observed as well as degenerative tubular changes. Immunofluorescence microscopy was positive only for fibrinogen (+3/+3) in vascular walls (Fig 1). Electron microscopy was not performed. The association of histological findings was compatible with TMA. Eculizumab therapy was initiated 15 days after the onset of symptoms. The symptoms evolved with worsening renal function, and RRT was required. A TGPS + MLPA was performed to investigate a genetic background associates do aHUS that identified a heterozygous gene variant in the CFI gene, c.209A>C, p.(Asn70Thr), a heterozygous gene variant in the CFB gene, c.1363G>A, p.(Val455Ile), and a heterozygous deletion encompassing the *CFHR1* and *CFHR3* genes.

The patient underwent RRT for another month after the start of eculizumab therapy, presenting recovery of renal function. He is currently on medication under outpatient follow-up.

### Case 3

The patient was a previously healthy 28-year-old male. He presented with fever, hypertension, vomiting, macroscopic hematuria, and reduced urine output, which began 6 days before his admission at the emergency department. On his physical examination, he had edema in the lower limbs 1+/4+, pallor 2+/4+, and jaundice 1+/4, and he was hypertensive (180 × 110 mm Hg). Laboratory tests showed hemoglobin 9.3 g/dl, 4,300 leukocytes/mm^3^, and 88,000 platelets/mm^3^, LDH 1.201U/L, total bilirubin 1.8 mg/mL, direct bilirubin 0.6 mg/mL and indirect bilirubin 1.2 mg/mL, normal total complement, C3, and C4; serum creatinine 3.5 mg/dL, urea 69 mg/dL, negative serology for HIV and hepatitis B and C; urinalysis: countless red blood cells, proteins 1+/4, 24-hour proteinuria 297 mg. IgG serology for dengue was negative, but that for IgM was positive. A renal biopsy was performed, revealing only 1 glomerulus at light macroscopy with no abnormalities. Focusing on vascular compartment, it was observed several arterioles with concentric hyperplasia and a luminal thrombus besides moderate interstitial fibrosis. Immunofluorescence microscopy was positive for IgM (+2/+3) and fibrinogen (+2/+3) in vascular walls (Fig 1). Electron microscopy was not performed. The association of histological findings was compatible with TMA. The patient was discharged from hospital with prescription of antihypertensive drugs. After 5 months, new laboratory tests showed remission of hematuria and proteinuria and partial recovery of kidney function (creatinine 2.5 mg/dL). The patient still remained hypertensive. He abandoned the outpatient follow-up, and, almost 2 years after the diagnosis, he returned to the emergency room for a dialysis emergency.

## Case discussion

TMA is characterized by thrombocytopenia, microangiopathic hemolytic anemia, and organ injury. It may manifest as a diversified spectrum of conditions, but AKI is a renowned aspect, due to a supposed predisposition of glomerular circulation to endothelial harm and occlusion [7].

In the cases above, 2 patients were diagnosed with dengue fever and 1 with chikungunya, based on epidemiology and positive ELISA IgM serologies against dengue and chikungunya viruses, respectively, in addition to TMA confirmed by kidney histopathology. The first 2 patients were diagnosed with aHUS, which is typified by a triad of microangiopathic hemolytic anemia, thrombocytopenia, and AKI, all presented by the patients.

ADAMTS-13, a specific cleaving protease of Von Willebrand factor, was measured in patients of cases 1 and 2, and it was moderately reduced (28%) and >100%, respectively. The lack of activity of this protease promotes the formation of microvascular platelet thrombi in small vessels. TTP usually presents with neurological signs, being renal involvement unusual [8]. Our 3 patients had severely alteration of renal function, in which 2 of them needed to start RRT. These data decreased the suspicion for TTP.

It is important to highlight that it is not always easy to differentiate between the icterohemorrhagic form of dengue and TMA associated with this viral infection, as happened with the patient in case 2. Jaundice accompanied by an excessive elevation of aspartate aminotransferase in patients affected by this disease should instigate the diagnosis of TMA [9].

In aHUS patients, TMA manifestations are an effect of dysregulation of the alternative complement pathway on the surface of cells. This alteration causes unchecked cell activity after complement activation by start factors, leading to secondary thrombosis, inflammation, and endothelial damage. A broad number of cases involve genetic and acquired factors [10]. It is postulated that, as in the *H1N1*-associated TMA, the arboviruses trigger TMA through direct endothelial damage, leading to platelet activation both directly and by exposing the extracellular matrix. Furthermore, in the pattern of the immune response to several viruses, there is activation of the alternative complement pathway, involved in the genesis of some TMA, especially aHUS [11–14].

The diagnosis of genetic aHUS is confirmed by the identification of pathogenic variants in 1 or more genes previously associated with genetic aHUS [15,16]. In almost 50% of aHUS patients, mutations in genes encoding complement regulating proteins are detected, resulting in either loss of function in a complement regulatory gene or in gain of function in an effector gene [17]. The Kidney Diseases Improving Global Outcomes (KDIGO) of aHUS and C3 glomerulopathy recommends that the genetic test used to investigate pathogenic variantes in aHUS and C3G must be accurate enough to detect copy number variation, hybrid genes, and other genomic rearrangements in the *CFH/CFHR*s genomic region. Those requirements must be fulfilled by the combination of a targeted gene panel plus MLPA or a whole exome or genome sequencing [12,15]. Both patients of cases 1 and 2 underwent to TGPS plus MLPA to aHUS, which were compatible to increased susceptibility to this condition, in the setting of the detection of multiple heterozygous variants. Although complete investigation was not performed in all the 3 patients such as stool culture and PCR in stool for Shiga toxin, there are other findings in favor of aHUS diagnosis in these patients. Enterohaemorrhagic *Escherichia coli* (EHEC)-associated HUS occurs primarily in children younger than 5 years of age and in the elderly. After an incubation period of 4 to 7 days, EHEC-infected patients develop diarrhea, and approximately 15% of cases develop HUS within an additional 2 to 10 days [18]. The age of presentation in the tree cases varied from 18 to 28 years old (young adults), and they had a shorter period of incubation (3 to 6 days) than expected to HUS.

When patients diagnosed with aHUS undergo genetic investigation, pathogenic variants are found in about 50% to 60% of cases. In cases with a positive family history, it has been estimated a penetrance of 50%. The contribution of genetics in the pathogenesis of aHUS occurs in 2 ways: the presence of a robust pathogenic variant that can trigger the disease by itself in a monogenic disease model, or, as a “2-hits model,” where the association of hypomorphic variants in the setting of environmental stimuli (such as viruses’ infections) can trigger the disease. In the cases described in this article that undergone genetic tests, we observed the presence of several heterozygous variants whose stimulation of arbovirus infection could have been triggered the disease. It is worthwhile to cite that approximately 50% of cases of aHUS are triggered by infections [19–21].

The presentation with AKI reflects the results of renal ischemia [7]. The patient in the first case began treatment with eculizumab 11 days after the first manifestations of aHUS. However, he developed AKI and required dialysis, a common manifestation of aHUS that is compatible with the literature survey (Table 1). The patient underwent RRT for approximately 90 days, when he presented recovery of kidney function.

**Table 1:** Demographic characteristics, treatment, and renal outcome in 11 patients with TMA related to dengue fever.

Author	Year	Sex	Age	Onset of symptoms (days)	Main diagnostic test	Variant	Treatment described	Outcome
Wiersinga and colleagues [22]	2006	Male	48	U	Kidney biopsy	aHUS	Plasmapheresis and hemodialysis	Lost track
Rossi and colleagues [23]	2010	Male	43	11	ADAMTS13 <10%	TTP	Plasmapheresis	Lost track
Hadianto and Mellyana [5]	2011	Male	8	4	Presumptive	aHUS	Clinical support hemodialysis	Lost track
Aroor and colleagues [6]	2014	Female	16	4	Presumptive*	aHUS	Plasmapheresis and hemodialysis	6-month follow-up: recovery of kidney
Deepanjali and colleagues [9]	2015	Female	25	2	Presumptive*	TTP	Plasmapheresis	Lost track
Bartholameuz and colleagues [24]	2016	Male	27	2	Presumptive*	TTP	Plasmapheresis	Lost track
Bhargava and colleagues [25]	2017	Male	32	U	Kidney biopsy	Undefined	Hemodialysis	9-month follow-up: recovery of kidney function
Gavali and colleagues [26]	2017	Female	35	7	Presumptive*	TTP	Plasmapheresis, GC, and RX	Lost track
Nieto-Ríos and colleagues [3]	2017	Male	21	7	ADAMTS13 normal, negative Shiga toxin kidney biopsy	Undefined	Plasmapheresis, GC, and hemodialysis	18-month follow-up: recovery of kidney function
Epelboin and colleagues [27]	2017	Female	43	4	ADAMTS13 <10%	TTP	FFP	Death
Bastos and colleagues [4]	2018	Male	28	U	ADAMTS13 <10%	TTP	Plasmapheresis and GC	Lost track

* Clinical diagnosis and response to treatment.

aHUS, atypical hemolytic uremic syndrome; FFP, fresh frozen plasma; GC, glucocorticoids; RX, rituximab; TMA, thrombotic microangiopathy; TTP, thrombotic thrombocytopenic purpura; U, uniformed.

In the second case, eculizumab therapy was introduced 15 days after the initial suspicion of aHUS. Eculizumab, a human monoclonal antibody that stops the activation of the C5 complement component and the final complement formation, has recently been shown to be effective in the treatment of aHUS [28–30]. It helps in the recovery of kidney function and can completely change the prognosis of this potentially catastrophic disorder [31].

In Brazil, eculizumab was registered by the National Health Surveillance Agency in 2017. However, this medication is not generally available for immediate use, and the import process may take 4 to 6 weeks. Therefore, in patients diagnosed with aHUS, standard treatment includes support measures, such as plasma infusion and plasmapheresis, until eculizumab is available [19]. It is important to highlight that the 2 patients diagnosed with aHUS received eculizumab quickly due to ease of donation at that time, which, in the authors’ opinion and based on publications about the medication, may have been decisive for the recovery of kidney activity in these patients. In the present study, the patient in case 1 presented serum creatinine of 1.52 mg/dL, and the patient in case 2 presented 1.15 mg/dL, which correspond to an estimated glomerular filtration rate by CKD-EPI formula of 65 mL/min/1.73 m^2^ and 81 mL/min/1.73 m^2^, respectively.

Ryan and colleagues conducted a retrospective study with 222 patients to examine the consequence of early introduction of eculizumab in aHUS. In that study, early administration was defined as happening in the first 7 days of hospitalization. The authors concluded that early administration of eculizumab was associated with a lower incidence of dialysis, shorter duration of stay in the intensive care unit, less need for plasmapheresis, and lower hospital expenses in comparison to the administration after 7 days [32].

There are some challenges and limitations related to the use of this medication in the treatment of aHUS. By inhibiting the formation of the membrane attack complex, patients under treatment are more vulnerable to developing infections by encapsulated microorganisms. Other challenges comprise the appropriate period of treatment and the excessive cost of the treatment with this drug [33].

The third case, despite having a diagnosis of dengue confirmed by IgM ELISA serology and evidence of TMA in the kidney histopathology, presented no criteria for either TTP or HUS/aHUS. One of the possibilities is that in this patient TMA was a consequence of malignant systemic arterial hypertension. This is characterized by very high blood pressure levels and multiple complications, such as severe organ damage, with poor prognosis. In addition, the coagulation system becomes activated, forming intravascular microthrombi and consequently causing hemolysis, which characterizes microangiopathic hemolytic anemia of malignant hypertension. This set of alterations leads to partial obstruction of small vessels and ischemia of different organs, particularly the kidney, where alterations are most evident [34].

Atypical manifestations of dengue infection may also involve the ability to form transient polyclonal antibodies directly against erythrocytes antigens, which, in turn, result in complement-mediated hemolysis [35].

There have been few reports of aHUS due to dengue virus infection and chikungunya. During the literature survey, 11 clinical cases were found whose patients had presented microangiopathic hemolytic anemia associated with dengue, and the majority of publications are from tropical or equatorial countries, because of the higher prevalence of dengue in these regions. Of those 11 cases, 6 were diagnosed with TTP, 3 with aHUS, and 2 with unclassified TMA. Of those patients, 7 either were lost to follow-up, or there was no report of their kidney function in the follow-up; 3 patients presented recovery of renal function, and there was 1 death (Table 1).

In conclusion, patients with arboviral infectious disease and changes that suggest TMA should have appropriate support in order to establish early diagnosis and useful treatment.

## Ethics statement

This study was approved by the Research Committee at the University Hospital, Federal University of Maranhão state. Written informed consent was obtained from every participant. All procedures involving human beings comply with the ethical standards of the Helsinki Declaration (1975/2008).

**Key Learning Points**

- Dengue fever and chikungunya are still health problems in developing countries.
- Thrombotic microangiopathy (TMA) is an uncommon manifestation of these arboviral infections.
- Acute kidney injury (AKI) is a renowned aspect of TMA.
- Eculizumab has been shown to be effective in the treatment of atypical hemolytic uremic syndrome (aHUS).

## Supporting information

S1 Table Reference ranges of laboratory tests. (DOCX)
