# Supplementary material for: Thrombotic microangiopathy associated with arboviral infection: Report of 3 cases
Source: PLoS Negl Trop Dis. 2021 Oct 14;15(10):e0009790. doi: 10.1371/journal.pntd.0009790 (PMC8516303; doi:10.1371/journal.pntd.0009790)
Supplement: S1 Table — (DOCX) [file pntd.0009790.s001.docx]

| **Laboratory Test** | **Reference Range** |
| --- | --- |
| ADAMTS 13 activity (%) | Severely decreased activity < 10% |
| Albumin (g/dL) | 3.5 - 5.0 |
| C3 (mg/dL) | 82 - 160 |
| C4 (mg/dL) | 12 - 36 |
| Urea (mg/dL) | 15 - 45 |
| Creatinine (mg/dL) | 0.6 - 1.3 |
| Estimated glomerular filtration rate by CKD-EPI (mL/min/1.73m^2^) | 110 - 150 |
| Protein/creatinine ratio from a single urine sample (mg/g) | < 150 mg / g |
| Hemoglobin (g/dL) | 13 - 16.9 |
| Hematocrit (%) | 39.7 - 52 |
| Lactate dehydrogenase (UI/L) | 135 - 214 |
| Leukocytes (mm^3^) | 3,600 - 11,000 |
| Platelets (mm^3^) | 150,000 – 300,000 |
| Sodium (meq/L) | 135 - 145 |
| Potassium (meq/L) | 3.5 - 5.5 |
| Prothrombin time (seconds) | 12 - 14 |
| Direct Bilirubin (mg/dL) | < 0,4 |
| Indirect Bilirubin (mg/dL) | < 0,8 |
| Total Bilirubin (mg/dL) | 0.3 - 1.2 |

**S1 Table. Reference ranges of laboratory tests**
